# Major bacterial isolate and antibiotic resistance from routine clinical samples in Southern Ethiopia

**DOI:** 10.1038/s41598-021-99272-2

**Published:** 2021-10-05

**Authors:** Mengistu Hailemariam, Tsegaye Alemayehu, Bereket Tadesse, Netsanete Nigussie, Asnakech Agegnehu, Techilo Habtemariam, Mulubrhan Ali, Enkosilassie Mitiku, Elshaday Azerefegne

**Affiliations:** 1 School of Medical Laboratory Science, Hawassa University College of Medicine and Health Sciences, P.O. Box 1560, Hawassa, Ethiopia; 2 Hawassa University Comprehensive and Specialized Hospital, Hawassa, Ethiopia

**Keywords:** Microbiology, Health care

## Abstract

Currently, antibiotic-resistant bacterial infections are a challenge for the health care system. Although physicians demand timely drug resistance data to guide empirical treatment, local data is rather scarce. Hence, this study performed a retrospective analysis of microbiological findings at the Hawassa public hospital. Secondary data were retrieved to assess the prevalence and level of drug resistance for the most common bacterial isolates from clinical samples processed at Hawassa University Comprehensive Specialized Hospital. Out of 1085 clinical samples processed in the microbiology laboratory, the prevalence of bacterial infection was 32.6%. Bacterial bloodstream infection was higher in children than in adults (OR, 4; 95% CI 1.8–14.6; *p* = 0.005). *E. coli* and *K. pneumoniae* were the commonest bacterial isolate both in children (36.8%, 26.3%) and in adults (33.3%, 26.7%) from the urine sample while, the leading bacteria identified from the CSF sample was *P. aeruginosa*, 37% in children and 43% in adult. In this study, all identified bacterial isolates were multi-drug resistant (MDR) ranging from 50 to 91%. The highest proportion of MDR was *S. aureus* 91.1 followed by *K. pneumoniae* 87.6%. Since the nationwide investigation of bacterial isolate, and drug resistance is rare in Ethiopia, a report from such type of local surveillance is highly useful to guide empirical therapy by providing awareness on the level resistance of isolates.

## Introduction

Bacterial infections and antibiotic resistant are threatening modern health care and have triggered the development of coordinated and comprehensive national and global actions plans. However, estimating the incidence, complications, and attributable mortality is challenging especially in Africa^1^. In developing countries like Ethiopia, the burden is so serious due to the unaffordability of the expensive alternative antibiotics for most of the patients^2^. The unregulated and often misusage of antibiotics provoked the development of antimicrobial resistance in the country^3^. Moreover, the rapid spread of antimicrobial-resistant organisms threatens the health care system due to a rising number of infectious diseases^4^.

Management of bacterial infection in Ethiopia has been largely empirical without the use of a bacterial culture and susceptibility testing to guide therapy. However, few teaching hospitals like Hawassa University comprehensive and specialized hospital (HU-CSH) have laboratory facilities to perform culture and antibacterial resistance tests. Indeed, this practice is a risk for development of antimicrobial resistance in most developing countries. In Ethiopia, where there are regulatory hindrances the challenge of antimicrobial resistance continued beside the scarcely available data^5^. The previous study has already reported the existence of elevated antimicrobial resistance (AMR) to different isolates, erythromycin (89.4%), amoxicillin (86.0%) and tetracycline (72.6%). The problem is more challenging due to about 75% of the burden found in developing countries^6^.

As to the WHOs declaration, AMR is one of the top 10 global public health threats facing humankind. Currently antibiotics are becoming increasingly ineffective as drug-resistance spreads globally leading to more difficult to treat infections. For common bacterial infections, including urinary tract infections, sepsis, sexually transmitted infections, and some forms of diarrhoea, high rates of resistance indicating that health facilities are running out of effective antibiotics^7,8^. There was considerable concern in several clinical situations where treatment options have become very limited and continues to threaten the ability to treat common infections^9^. In low-income countries, like Ethiopia, the problem is higher due to misuse and overuse of antimicrobials in addition to lack of laboratory facilities for antimicrobial susceptibility testing^10^.

According to the World Health Organization (WHO), bacterial resistance to first-line drugs ranges from zero to almost 100%. In some instances, resistance to second-and third-line drugs seriously compromising treatment outcome^11^. Indeed, due to the complex effect of drug resistance, it became global attention that needs urgent action to improve and coordinated global effort to encompass AMR^12^.

Infections by ESKAPE (*Enterococcus*, *S. aureus*, *K. pneumoniae*, *A. baumannii*, *P. aeruginosa*, and *E. coli*) organisms are the leading cause of healthcare-acquired infections worldwide. Most of them are multidrug resistant isolates, which is one of the greatest challenges in clinical practice^13,14^. It has been said also that, ESKAPE pathogens have developed resistance mechanisms either through inactivation or alteration of the antimicrobial molecule, bacterial target site modifications, reduced antibiotic penetration/accumulation, or by formation of bacterial biofilms^14,15^. The acquisition of antimicrobial resistance genes by ESKAPE pathogens has reduced the treatment options for serious infections^15,16^.

*Enterococcus faecium* is a prominent cause of health care-associated infections, several countries have now reported increases in resistance in hospitalized patients^14,17,18^. Methicillin-resistant staphylococci (MRSA) are also a major causes of bacterial infections with a serious challenge of antibiotics resistance^19^. The mecA gene, encoding MERSA, is located on a mobile genetic element called Staphylococcal Cassette Chromosome mec (SCCmec). Horizontal, interspecies transfer of this element could be an important factor in the dissemination of MRSA^20,21^. Many antibiotics, including β-lactams, can induce the expression of SCCmec excision from the bacterial chromosome^22^.

On the other hand, cephalosporin resistance due the enzymes β-lactamase, extended-spectrum lactamases (ESBLs) and carbapenemases destroys the β-lactam antibiotics such as penicillin, cephalosporins, and carbapenem drugs^15,23,24^. *A. baumannii and E. coli* also resist a number of carbapenem and lactam class antibiotics^25,26^. Whereas *P. aeruginosa*, the common infections in patients with impaired immunity^27,28^ more and more resisting antibiotics through intrinsic and acquired resistance mechanisms^29^.

The governmental organization known as Ethiopian Food, Medicine and Health Care Administration and Control Authority (EFMHACA) bears the responsibility to regulate drugs, food, health care personnel and setting at the central level. The authority controls and regulates drugs used in the country^30^. However, data’s on rates of bacterial isolate and AMR are scarce therefore, the local survey of laboratory data to note major isolates and their antibiotic resistance were as important to guide optimal empiric treatment of patients^31^.

Thus, the aim of this study is to indicate major pathogenic isolates and their antimicrobial resistance in the study area so that the finding supports health professionals empirical therapy.

## Methods

A retrospective cross-sectional study was conducted at the microbiology laboratory of HUCSH, Hawassa, Ethiopia. HU-CSH is a teaching hospital providing services to more than 5 million people in the region as well from nearby region. Likewise, the HU-CSH microbiology laboratory also responsible for bacterial culture and resistance tests for patients in the area. The laboratory was supervised and supported by Federal government through the Ethiopian public health institute (EPHI). The EPHI makes mentor and monthly technical support including external quality assessment.

Samples were collected by the physician or the nurses from all inpatient and outpatient departments and immediately sent to the microbiology laboratory. The decision to take samples for microbiological culture and the selection of samples was made by the physicians. While, patient-related data (age and sex) with a full record of bacteriological culture and antimicrobial resistance profile were retrieved from the laboratory registration book. The study retrieved all microbiological reports on bacterial pathogens from January 2019 to December 2020.

### Study participants and data collection

The study participants were individuals who visit HU-CSH and had complain of any infection suspected for bacterial infections during the study period. This study retrieved all documented data’s including age and sex of patients, the bacteria isolated and the drug susceptibility profiles from HU-CSH microbiology unit registration books using a standard data collection form.

All laboratory procedures were performed in accordance with the relevant guidelines and regulations of the Clinical and Laboratory Standards Institute’s (CLSI)^32^ and accustomed to the local standard operation procedures (SOPs) of our microbiology unit. Data obtained in the course of the study were kept confidential and used only for this study. We included all documented data within the study period except we rejected illegible or incompletely documented culture results.

### Bacteriological investigation

Our bacteriological analysis follows SOPs adopted from CLSI guideline. The collected clinical samples were submitted to the laboratory and processed following standard procedures. Several types of clinical samples were cultured, including stool, urine, blood, urethral smear, and swabs from various body sites (vagina, ear, eye and wound). Each clinical samples employed similar standard microbiological culturing techniques. Specimens collected were inoculated onto appropriate isolation culture media and incubated at 35–37 °C conferring to standard protocols for each sample^33^. Bacterial identification was done primarily based on colony characteristics and Gram-stain reaction followed by proper biochemical tests as per suitability according to CLSI guidelines and developed SOP.

### Antimicrobial susceptibility

The antimicrobial susceptibility profile of isolates was determined by Kirby-Bauer disc diffusion method and the results were interpreted according to CLSI guidelines^34^. The antimicrobial discs were obtained from Oxoid (Oxoid, Hampshire, UK) in the following concentrations: Amoxicillin-Calvulanic acid (AMC, 20/10 μg), cotrimoxazole (SXT, 25 μg), Ceftazidime (CAZ, 30 μg), cefotaxime (CTX, 30 μg), Chloramphenicol (CAF, 30 μg), nitrofurantoin (NIT 300 μg), ceftriaxone (CRT, 30 μg), ampicillin (AMP,10 μg) Erythromycin(E, 30 μg), gentamicin (*GN*, 10 μg), meropenem (MER 10 μg), Amikacin(AMK, 30 μg), ceftriaxone (CRO, 30 μg). The definition of MDR in this study refers to resistant to at least one agent in three or more antimicrobial classes.

### Quality control

The quality of our laboratory culture system was performed using *E. coli* (ATCC-25922), *P. aeruginosa* (ATCC-27853), and *S. aureus* (ATCC-25923) as reference strains for culture and susceptibility testing. Thus, qualities of prepared media were monitored all the time according to the set criteria by CLSI in fact EPHI closely supervised our laboratory monthly.

### Data analysis

The data was entered and analysed using SPSS version 20 (IBM Corporation, Armonk, NY, USA). Descriptive statistics like frequency and percentages of categorical variables were calculated. Bivariate comparisons using Chi-square logistic regression were employed to assess the association between variables. A p-value of less than 0.05 was considered statistical significant.

### Ethical clearance

Ethical clearance was obtained from the institutional review board (IRB) of Hawassa University College of medicine and health science. As the study was retrospective, following IRB clearance, we have asked the hospital administration for a waiver to retrieve the data. Accordingly, the official waiver letter was obtained from the HU-CSH hospital administration. We have also obtained official permission from the hospital laboratory manager of HU-CSH. All data obtained in the course of the study were reserved confidential and used only for this study.

## Result

### Study population

In this study, a total of 1085 clinical samples were processed. Of these, females were one-third 385 (35.5%) of the study population. Likewise, the rate of positivity in female is lower 114 (32.2%) compared to males 240 (67.8%) but not significantly associated (*p* = 0.11). Regarding the age group, the proportion of under-5 children was about two-thirds (61.3%) of participants. There was a significant association between age group of participants and positivity of bacterial infection (*p* = 0.154). The age range of participants was from zero-day neonates to 82 years old elders with a mean age of 8.7 years (Table 1).

**Table 1:** Age and sex distribution of patients with rate of infection.

Age and sex	Positive n (%)	Negative n (%)	Total n (%)	X2 (*p* value)
**Sex**				
Male	240 (67.8)	460 (62.9)	700 (64.5)	2.5 (0.11)
Female	114 (32.2)	271 (37.1)	385 (35.5)	
**Age in years**				
0–4	233 (65.8)	432 (59.1)	665 (61.3)	
5–9	28 (7.9)	81 (11.1)	109 (10.0)	
10–19	45 (12.7)	98 (13.4)	143 (13.2)	8 (0.154)
20–29	18 (5.1)	61 (8.3)	79 (7.3)	
30–39	10 (2.8)	23 (3.1)	33 (3.0)	
> 40	20 (5.6)	36 (4.9)	56 (5.2)	
Total	354 (32.6)	731 (67.4)	1085 (100)	

Of 1085 clinical sample diagnosed in microbiology laboratory 32.6% (354/1085) of them were positive for any of bacterial infection. The proportion of positivity was decrease with age (Table 1).

### Bacterial spectrum

The overall prevalence of the bacterial infection was (354/ 1085, 32.6%). The predominant type of infection was bloodstream infection (78, 22%). Of the total 354 identified isolates, 91 were from urine, 34 from CSF, 105 from blood and the remaining 91 were documented from different clinical samples (including pus, discharge and stool sputum) (Table 3). Nearly 46% (n = 162) of the isolate were attained from patients those who have one or two types of the previous history of antibiotic exposure before specimen collection.

The children were more positive in bloodstream infection than adults (OR, 4; 95% CI 1.8–14.6; *p* = 0.005) (Table 2). The isolates from different groups of infections were not similar however, common bacterial pathogens were documented in both adults and children (Tables 2, 3). For instance, nearly equal proportions of *E. coli* and *K. pneumoniae* isolates were observed in children (36.8%, 26.3%) and adults (33.3%, 26.7%) from urine samples. Again, a comparable result was recorded for *P. aeruginosa* in the CSF sample, 37% in children and 43% in adults. On the other hand, the most frequent isolate from blood cultures in children was CoNS (29.5%) followed by *K. pneumoniae* (25.7%) and *S. aureus* (20%). But in adult CoNS (60%) followed by *S. aureus* (20%) were found (Table 3).

**Table 2:** Distribution of isolates from various infections in children and adult patients.

	Children, n (%)	Adults, n (%)	OR (95% CI)	*p* value
Urinary tract infection	76 (34)	15 (30.3)	1.2 (0.6–2.3)	0.6
CSF infection	27 (9.3)	7 (29.2)	4 (1.5–10.5)	0.005
Bloodstream infection	105 (61.4)	5 (23.8)	5.1 (1.8–14.6)	0.002
Others	95 (43.0)	30 (34.9)	1.4 (0.85–2.3)	0.19
Total	299 (100)	55 (100)	–	–

**Table 3:** Isolates from main infection sits in children and adults.

Infection	Pathogen	Children, n (%)	Adults, n (%)	Total, n (%)
UTI	*E. coli*	28/76 (36.8)	5/15 (33.3)	33/91 (36.3)
	*K. pneumoniae*	20 (26.3)	4 (26.7)	24 (26.4)
	*Proteus mirabilis*	8 (10.6)	1 (6.7)	9 (9)
	Others	20 (26.3)	5 (33.3)	25 (27.3)
CSF infection	*P. aeruginosa*	10/27 (37)	3/7 (42.8)	13/34 (38.2)
	*K. pneumoniae*	9 (33)	1 (14.3)	10 (29.4)
	*Acintobacter*	4 (15)	2 (28.6)	6 (17.6)
	Others	4 (15)	1 (14.3)	5 (14.8)
Bloodstream infection	*CoNS*	31/105 (29.5)	3/5 (60.0)	34/110 (31)
	*K. pneumoniae*	27 (25.7)	–	27 (24.5)
	*S. aureus*	21 (20)	1 (20)	22 (20)
	Others	26 (24.8)	1 (20)	27 (24.5)
Others	*S. aureus*	22/91 (24.2)	10/28 (35.7)	32/119 (26.8)
	*K. pneumoniae*	15 (16.4)	6 (21.4)	21 (17.5)
	*E. coli*	13 (14.3)	2 (7.1)	15 (12.5)
	CONS	10 (11.1)	3 (10.7)	13 (11)
	Others	31 (34)	7 (25.2)	38 (32)
Total		299 (84.5)	55 (15.5)	354 (100)

### Antibiotic resistance

As indicated in Table 4, most of the gram positive isolates showed the highest resistance to erythromycin and gentamicin for instance; *S. pyogens* (75%), *S. aureus* (55%). CoNS showed about 30–55% resistances to Trimethoprim-Sulphametoxazole, ceftazidime, erythromycin and gentamicin. The *Enterococci* spp. also showed 85.7% resistance to gentamicin and 57.2% to Trimethoprim-Sulphametoxazole, respectively.

**Table 4:** Non-susceptibility rates of the gram positive bacteria.

Pathogen (n)	Antimicrobial non-susceptibility (%)											
	AMC	SXT	CAZ	CTX	CAF	NIT	CRT	E	AMP	GN	MER	AMK
*S. aureus* (56)	7 (12.5)	17 (30.4)	18 (32.2)	3 (5.3)	14 (25)	6 (10.7)	19 (34)	29 (51.8)	37 (66)	31 (55.6)	3 (5.3)	12 (21.4)
*CoNS* (48)	14 (29.2)	22 (45.8)	11 (22.9)	7 (14.6)	20 (41.7	6 (12.5)	1 (2.1)	24 (50)	31 (64.5)	20 (41.7)	6 (12.5)	9 (18.7)
*Enterococci* spp n = 7	1 (14.5)	4 (57.2)	4 (57.2)	4 (57.2)	2 (28.5)	2 (28.5)	1 (14.5)	4 (57.2)	4 (57.2)	6 (85.7)	1 (14.2)	2 (28.5)
*S. pyogens* (n = 4)	2 (50)	2 (50)	1 (25)	1 (25)	3 (75)	0	1 (25)	3 (75)	–	3 (75)	0	1 (25)

*CoNS*, Coagulase Negative Staphylococci; AMC, Amoxicillin-Calvulanic acid (20/10 μg); SXT, cotrimoxazole (25 μg); CAZ, Ceftazidime (30 μg); CTX, cefotaxime(30 μg); CAF, Chloramaphenicol; NIT, nitrofurantoin (300 μg); CRT, ceftriaxone (30 μg); AMP, ampicillin (10 μg); E, Erythromycin (30 μg); GN, gentamicin (10 μg); MER, meropenem (10 μg); AMK, Amikacin (30 μg).

Among gram-negative bacteria, *K. pneumoniae* has shown the highest resistance rates to ampicillin (75%), ceftazidime (82%) and ciprofloxacin (80.9) while the least resistance (3.3%) to meropenem was documented. Likewise, *E. coli* also showed the highest resistance to cotrimoxazole (70.4%) and ciprofloxacin (63.6%) while less resistant to meropenem (4.5%). In general, in this study gram-negative isolates were susceptible to meropenem (Table 5).

**Table 5:** Non-susceptibility rates of the most prevalent gram negative bacteria.

Pathogen (n)	Antimicrobial non-susceptibility (%)										
	AMP	AMC	AMK	CAZ	CTX	SXT	CAF	CRO	GN	MER	CIP
*E. coli* (52)	33 (63.5)	18 (34.6)	20 (38.5)	21 (40.4)	8 (15.4)	31 (59.6)	14 (26.9)	14 (26.9)	22 (42.6)	2 (3.8)	28 (53.4)
*K. pneumoniae* (89)	76 (85.4)	52 (58.4)	56 (63)	73 (82)	24 (27)	46 (51.6)	19 (21.3)	59 (66.3)	19 (21.3)	3 (3.3)	72 (80.9)
*P. aeruginosa* (35)	29 (82.9)	24 (68.6)	18 (51.4)	6 (17)	21 (60)	12 (34.3)	9 (25.7)	13 (37.1)	4 (11.4)	3 (8.6)	24 (68.6)
*Seratia spp* (8)	–	6 (75)	5 (62.5)	3 (37.5)	5 (62.5)	2 (25)	6 (75)	6 (75)	2 (25)	0	6 (75)
*Proteus mirabilis* (11)	4 (36.4)	8 (72.7)	4 (36.4)	5 (45.5)	6 (54.5)	5 (45.5)	3 (27.7)	5 (45.5)	5 (45.5)	1 (9)	4 (36.4)
Acintobacter spp (17)	–	8 (47)	9 (52.9)	15 (88)	8 (47)	5 (29.4)	6 (35.3)	6 (35.3)	11 (64.7)	3 (17.6)	13 (76.5)

AMP, ampicillin (10 μg); AMC, Amoxicillin-Calvulanic acid (20/10 μg); AMK, Amikacin(30 μg); CAZ, Ceftazidime (30 μg); CTX, cefotaxime(30 μg); SXT, cotrimoxazole (25 μg); CAF, Chloramphenicol; CRO, ceftriaxone (30 μg); GN, gentamicin (10 μg); MER, meropenem (10 μg); CIP, Ciprofloxacin (5 μg).

### Multidrug‑resistance profiles of the isolates

As indicated in Table 6, *S. aureus* and *K. pneumoniae* documented a highest multidrug-resistance for more than two antibiotics, 91.1% (51/56) and 87.6% (78/89) respectively. About 15% of *S. aureus* isolates were resisted to more than 5 antibiotics while only 5.4% of them were susceptible to tested drugs. Likewise, 15% *K. pneumoniae* isolates resisted for more than 5 antibiotics and only 4.5% susceptibility was documented. According to our finding, 9 (16%) of *S. aureus* were resistant to AMC, SXT, CAF, CRT, GN and CAZ. Around 6 (10%) isolates were also resistance to CAZ, CTX, and CAF. Similarly, 15 (28.8%) of *E. coli* were resistant to AMP, AMC, AMK, SXT, CAF and CIP, while 3 (5.8%) for CAZ, CIP and AMK.

**Table 6:** Antimicrobial resistance pattern of common pathogenic bacteria isolate from different sites of infections.

Bacterial isolates	R0	R1	R2	R3	R4	R5	R > 5	Total MDR
*K. pneumoniae* (89)	4 (4.5)	7 (1.1)	15 (16.8)	23 (25.8)	19 (21.3)	8 (8.9)	13 (14.6)	78 (87.6)
*S. aureus* (56)	3 (5.4)	2 (3.6)	12 (21.4)	6 (10.7)	13 (25)	11 (23.2)	9 (16.1)	51 (91.1)
*E. coli* (52)	5 (9.6)	8 (15.4)	6 (11.5)	3 (5.8)	1 (1.9)	14 (26.9)	15 (28.8)	39 (75)
CoNS (48)	6 (12.5)	5 (10.4)	7 (14.6)	11 (22.9)	6 (12.5)	7 (14.6)	6 (12.5)	37 (77.1)
*P. aeruginosa* (35)	4 (11.4)	6 (17.1)	4 (11.4)	5 (14.3)	10 (28.6)	4 (11.4)	2 (5.7)	25 (71.4)
*Acintobacter* spp. (17)	2 (11.8)	3 (17.6)	2 (11.8)	3 (17.6)	6 (35.3)	1 (5.9)	0	12 (70.6)
*Proteus mirabilis* (11)	1 (9.1)	3 (27.3)	1 (9.1)	1 (9.1)	3 (27.3)	1 (9.1)	1 (9.1)	7 (63.6)
*Seratia* spp. (8)	0	3 (37.5)	1 (12.5)	3 (37.5)	1 (12.5)	0	0	5 (62.5)
*Enterococci* spp. n = 7	2 (28.6)	1 (14.3)	2 (28.6)	1 (14.3)	0	1 (14.3)	0	5 (71.4)
*S. pyogens* (n = 4)	1 (25)	1 (25)	0	0	2 (50)	0	0	2 (50)

R0: susceptible to all antimicrobials tested; R1, R2, R3, R4, R5, R > 5: Resistance to one, two, three, four, five, and more than five antimicrobials, respectively.

## Discussion

The burden of drug-resistant bacterial infections remains a global challenge that triggers the development of global actions. According to this study, the leading bacterial isolates were *K. pneumoniae* 89 (25.2%) and *S. aureus* 56 (15.8%). Out of 1085 clinical samples processed, 32.6% of them were culture positive of which the gram-negative pathogens were dominant. It is similar to others report that *K. pneumoniae* and *S. aureus* were dominant isolates from different clinical samples^35–37^.

According to this study, the major bacterial isolate in bloodstream infection among children is CoNS and *K. pneumoniae* which is similar to our previous report^38^ and with others finding at northern^39^ and central Ethiopia^40–42^. Nearly similar to a study in Egypt, Gabon and Zimbabwe where CoNS*, E. coli* and *K. pneumoniae* were the major isolates in bloodstream infection^43–45^. In our finding, *P. aeruginosa* and *K. pneumoniae* are the major bacteria isolated from CSF unlike others report^5,38,41,46^. On the other hand, study from Tanzania has reported that *S. aureus* was the main pathogen in bloodstream infection unlike our finding^47^. Based on this finding, CoNS was the major pathogen in bloodstream infection. The possible attribute might be the poor hygienic care as well as the higher nosocomial infection in our setup^48^ that significantly contributes to the high proportion of CoNS. A recent report also showed the highest prevalence of hospital-acquired bacterial infections in a developing country^49^.

In regard to the isolates from UTI, *E. coli* and *K. pneumoniae* were documented as major bacterial agent which is consistent with a study conducted with others^41,50^. Our finding was slightly higher than previously published results in Ethiopia^35,36^. Of course, the variation in bacterial isolate of UTI infection has been seen across region^50^. This could be due to differences among study participants, catheterization and hospitalization history.

Unlike our previous report, where *N. meningitis* and *E. coli* were the *pre*dominant isolate in CSF^38^, in this study *P. aeruginosa and K. pneumoniae* were the leading isolate. In this study the highest number of bacterial infection were documented in neonates which shows that newborns might be at higher risk of bacterial infection. Hence adequate attention should be given particularly in maintaining the proper hygiene at the ICU^51^.

From other specimens like stool, pus, sputum and discharges, *S. aureus* and *K. pneumoniae* were found a major infectious agent. Compiling such type of laboratory database is useful to provide valuable insight about the types of sample and identified drug resistant isolates in areas where microbiological investigations are scarce^41^. Hence our data can be used in support of the existing empirical therapy.

Now a day, MDR bacterial infections due to ESKAPE groups become a major health problem and account for a great burden in the effective therapeutic strategies^52^. Our study showed high-level ESKAPE groups MDR (greater than 70%) which is comparable with others report in Ethiopia^41,42^. However, certain variation in bacterial isolate and resistance level reported within-country^5,39^ besides related reports in Africa^43,45^. This might be due to a lack of consistency in the measurement and reporting of drug susceptibility data. In fact, most studies were conducted using a disk diffusion technique similar to this study^53^.

The rate of overall MDR *S. aureus* documented in this study is similar to most other studies^54–59^. This might be due to the fact that *S. aurous* can present and cause infection to diverse sites of a human body beyond being normal commensal. The major antibiotics prescribed for *S. aurous* in local area were mostly resisted emphasizing the need for continuous investigation as well as etiologic based therapy. However, in Ethiopia majorities of hospitals were not performing bacteriological culture routinely. Hence, clinician need to look for such type of compiled reports for further consideration of their antibiotics selection.

On the other hand, nearly 88% of *K. pneumoniae,* 75% *E. coli* and 70% of *P. aeruginosa* isolates were resistant to the tested antibiotics. These isolates were mainly identified as hospital-acquired pathogen also they were reported to be community-acquired. The ESKAPE pathogens are the leading cause of nosocomial infections and most of them are MDR, which is one of the greatest challenges in clinical practice amongst critically ill and immunocompromised individuals^15^. Our finding is somehow comparable to previous studies where MDR-ESKAPE reported as predominant isolates^55,60–62^. The resistances acquired to available drugs either by mutation or receipt of foreign genetic materials through the transfer of plasmids and transposons of other microorganisms of the surrounding flora.

In general, the Gram-negative ESKAPE pathogens drug resistance in this study was somehow comparable with others finding for instance, with a study in Latin American countries^63^, Sudan^64^ Northern Ethiopia^9^ and the systematic reviews conducted in Ethiopia^65,66^. According to the systematic review, the Gram-negative ESKAPE pathogens were highly drug-resistant with estimates between 76 and 87%^66^. Similarly, high MDR *Acinetobacter* isolates usually reported around 45% of hospitalized patients globally^14^. In Asia–Pacific countries, around 57% of *A. baumannii* were resistant to all tested drugs^67^. While our finding showed a bit higher, 70% that is similar with Latin American countries report^63^.

Such type of study has an important effect to help the approval of guidelines to the empirical choice of antibiotic therapy. Moreover, from the patient perspective, an appropriate antibiotic resistance profile is essential to reduce mortality and length of hospitalization, the loss of work and family time associated with increased hospitalization time and subsequent recovery, and even the emotional impact of having a resistant infection. In fact, the economic burden of antimicrobial resistance in the prospect of hospital costs, hospital charges including operation costs, the cost of drugs and tests, and other patient care activities and resources used is also a major concern. Hence, such types of studies have a great role for clinicians as well as for policy makers^68^.

### Limitations

There were certain limitations in this study: First, this retrospective study does not includes all patients visiting our hospital due to some the fact that in our setup culture is not ordered for non-critical patients. For instant most asymptomatic STI and enteric infections by default managed by empirical therapy. Second, we can’t compare our findings by dwelling and wards as these variables were not registered in laboratory log book Third, we did not investigate risk factors for infection as well as for drug resistance as it is retrospective data.

## Conclusions

According to our investigation, a high rate of bacterial infection (32.6%) was reported and most of the isolates resist the commonly used antibiotics. *K. pneumoniae*, *S. aureus*, *E. coli* were the common isolates in most clinical samples meanwhile CoNS was major bloodstream infection in neonates. The least resistance antibiotics for gram-positive isolate were cefotaxime, meropenem nitrofurantoin and amoxicillin-calvulanic acid. On the other hand, highest levels of resistance were seen for ciprofloxacin, ampicillin and cotrimoxazole. We advise clinicians to see options from such type of compiled data for their routine empirical therapy of bacterial infections.

## Data Availability

The data that support the findings of this study are available from the corresponding author on upon reasonable request.

## Abbreviations

AMR: Antimicrobial resistance
ATCC: American type culture collection
CLSI: Clinical and laboratory standard Institute
CoNS: Coagulase-negative staphylococcus
CSF: Cerebrospinal fluid
HUCSH: Hawassa University Comprehensive Specialized Hospital
IRB: Institutional Review Board
SNNPR: Southern Nation Nationalities’ and peoples’ region
SOP: Standard operating procedure
SPSS: Statistical package for social sciences
WHO: World Health Organisation
